# Suppression of Proinflammatory Cytokines by *Etlingera alba* (A.D.) Poulsen Rhizome Extract and Its Antibacterial Properties

**DOI:** 10.1155/2021/5570073

**Published:** 2021-06-17

**Authors:** Rini Hamsidi, Wahyuni Wahyuni, Idin Sahidin, Evi Apriyani, Harsono Harsono, Nur Arsianti Azizah, Fadhliyah Malik, Agung Wibawa Mahatva Yodha, La Ode Muhammad Julian Purnama, Adryan Fristiohady

**Affiliations:** ^1^Department of Health, Faculty of Vocational Studies, Universitas Airlangga, Surabaya 60286, Indonesia; ^2^Department of Pharmacy, Faculty of Pharmacy, Halu Oleo University, Kendari, Southeast Sulawesi, Indonesia; ^3^Department of Pharmacy, Polytechnic of Bina Husada, Kendari, Southeast Sulawesi, Indonesia; ^4^Thammasat University Research Unit in Drug, Health Product Development and Application (DHP-DA), Department of Pharmaceutical Sciences, Faculty of Pharmacy, Thammasat University, Khlong Luang, Pathum Thani 12120, Thailand

## Abstract

*Etlingera alba* is one of the *Etlingera* plants that has not been studied intensively. Plants that belong to the same genus have similar constituents and pharmacological activities. Thus, we aim to investigate the chemical composition and pharmacological activities, namely, anti-inflammatory and antibacterial properties, of *E. alba* rhizome extract (EA). The chemical constituent was detected using the test tube method. The inflammatory model rats were obtained by inducing them with 1% carrageenan, and their palm edema volume and cytokine levels, namely, IL-6, IL-12, and TNF-*α*, were measured. Antibacterial activity was performed with broth microdilution. The phytochemical screening of EA was detecting alkaloids, flavonoids, tannins, steroids, and phenols. The EA has anti-inflammatory activity by reducing the palms' edema volume and cytokine levels (IL-6, IL-12, and TNF-*α*), and the optimal concentration was 400 mg/kg body weight (BW). On the other hand, EA also exhibited antibacterial properties against *E. coli* and *S. enterica.* In conclusion, similar to other *Etlingera* plants, EA also demonstrates pharmacological activities, namely, anti-inflammatory and antibacterial properties.

## 1. Introduction

Indonesia is a country that has the second highest biodiversity in the world after Brazil. Of the 40,000 species globally, 30,000 are found in Indonesia, and 940 are known to have medicinal properties. The number of medicinal plants covers around 90% of the total medicinal plants found in the Asian region [1]. Utilization of medicinal plants in the ethnobotany studies is an effective way of finding new chemicals useful for treatment. One type of the plants widely used in traditional medicine is a plant from the *Etlingera* genus [2, 3].

Empirically, the *Etlingera* plants are widely used as spices as well as herbal medicine. For example, the *Etlingera elatior* is mainly used as a flavor enhancer, food preservative, wound and earache medicine, and deodorant [4]. Besides, *E. brevilabrum* has been used to treat dry skin, fever, sore eyes, and stomachache, as well as condiments [5]. Because of this, many plants from *Etlingera* have potency as a source for compounds in drug searching.

The pharmacological activity of plants from the genus *Etlingera* has been widely reported. *Etlingera elatior* has activity as an antioxidant, anti-inflammatory, immunomodulatory, anticancer, and antibacterial. It also provides nephroprotective and hepatoprotective activity [6–11]. *E. brevilabrum* leaves and stem are reported to decrease cholesterol levels [12]. On the other hand, *E. calophrys* and *E. fulgens* have antibacterial activity and free radicals' scavengers. *E. calophrys* also provides antidiabetic activity [13, 14]. The agents that are responsible for providing these properties are mainly flavonoids (quercetin, apigenin, kaempferol, luteolin, and myricetin) and phenolic compounds (gallic acid, tannic acid, chlorogenic acid, and caffeic acid) due to their antioxidant properties [15].

One species of *Etlingera* that has never been explored much before is *Etlingera alba*. Various studies suggest that plants belonging to the same genus usually have similar activities. *E. alba* can contain anti-inflammatory and antibacterial activities by reflecting on research conducted on other *Etlingera* genus plants. A recent study performed showed that *E. alba* has antibacterial activity [16]. Thus, the study investigated the anti-inflammatory by measuring the edema volume and the cytokines involved in inflammatory process, including IL-6, IL-12, and TNF-*α*. The study also investigated the antibacterial activity of the ethanol extract of *E. alba* rhizome using microdilution method.

## 2. Materials and Methods

### 2.1. Sample Collection, Determination, and Preparation

The sample used was *E. alba* (Blume) A.D. Poulsen Rhizome was obtained from Punggaluku, South Konawe Regency, Southeast Sulawesi. Biology of Research Centre of LIPI was determining the sample (no. 1535/IPH.1.01/If.07/VIII/2019). The sample (20 kg) was wetly sorted, followed by chopping, drying, and dry sorting, continued by powdering the sample and putting in a sealed jar.

### 2.2. Extraction

Powdered sample (2.1 kg) was macerated with 96% ethanol for 3 × 24 hrs. The filtrate obtained was concentrated using a rotary vacuum evaporator at 40°C (90 rpm). The concentrated extract yielded was 94 g (4.47%).

### 2.3. Phytochemical Screening

Phytochemical screening of *E. alba* rhizome was performed qualitatively by colorimetric methods according to the modified Harborne method to detect alkaloids, flavonoids, steroids/terpenoids, tannins, phenols, and saponins [17].

#### 2.3.1. Alkaloid

The crude extract was mixed with ethanol and put in tube. The mixed extract was added with Dragendorff reagent. The presence of alkaloids was characterized by forming brown sediment.

#### 2.3.2. Flavonoid

The extract solution was mixed Mg. And then, the mixture was added with 1 mL of HCl. The discoloration into red, orange, yellow, and green indicates the presence of flavonoids.

#### 2.3.3. Steroid/Terpenoid

The extract solution was mixed with 1 mL of CH_3_COOH and 1 mL of H_2_SO_4_. The reddish brown-ring formed between solvents indicated the presence of terpenoids, while blue or greenish ring formed indicated the steroids.

#### 2.3.4. Tannin

The crude extract was mixed with ethanol and added with 3 drops of 5% FeCl_3_. The presence of tannin was indicated by discoloration of mixture into blackish green or navy.

#### 2.3.5. Phenols

The extract solution was mixed with 2 mL of 2% FeCl_3_ solution. A turquoise or black color indicates the presence of phenols. The discoloration into green, blue, or purple indicates the phenolic compounds.

#### 2.3.6. Saponin

The crude extract was mixed with 10 mL of distilled water in a tube. After that, the tube was vigorously vortexed until producing a foam. The foam formed indicated the presence of saponins.

### 2.4. Animals

Animals used were Wistar male rats obtained from an animal farm in Surabaya, West Java, Indonesia. The animals were acclimatized for 2 weeks in a standard environment (25 ± 1°C, Rh 55 ± 5%, and 12 : 12 h light/dark cycle). Animals were accessed with food and water *ad libitum.* After acclimatization, animals (*n* = 24) were divided into 6 groups (*n* = 4), which were Groups I–VI. Each group was given an induced 1% carrageenan (100 *μ*l) intraplantar, except Group VI, a normal group.

### 2.5. Anti-Inflammatory Assay

#### 2.5.1. Anti-Inflammatory Model

Each animal was induced with 1% carrageenan on the hind paw obtained inflammatory model rats. 1 hr after being induced with 1% carrageenan, all animals were treated orally as follows:

  Group I: 0.5% Na CMC as negative control
  Group II: ethanolic extract of *E. alba* rhizome dose of 200 mg/kgBW (EA200)
  Group III: ethanolic extract of *E. alba* rhizome dose of 300 mg/kgBW (EA300)
  Group IV: ethanolic extract of *E. alba* rhizome dose of 400 mg/kgBW (EA400)
  Group V: sodium diclofenac as the positive control
  Group VI: the group that received any treatment as normal control

#### 2.5.2. Edema Volume Measurement

1 hr after being induced with 1% carrageenan, all animals in Groups I–V were measured with their palm's volume, pre- (*V*_0_) and post-induced (*Vt*) in a plethysmometer. The edema volume differed between *Vt* and *V*_0_ in percentage [18].

#### 2.5.3. Cytokines Level Measurement

1 hr after treatment, the blood was collected by cardiac puncture and put in EDTA-tube in all groups. The collected blood was centrifuged at 3000 rpm for 15 minutes and continued by assaying the blood with Rat IL-6 ELISA Kit for plasma IL-6, Rat IL-12 ELISA Kit for plasma IL-12, and Rat TNF-*α* ELISA Kit for TNF-*α* levels.

#### 2.5.4. Statistical Analysis

Data collected was analyzed statistically using SPSS to examine EA's effect on the levels of cytokines (Il-6, IL-12, and TNF-*α*) by using one-way ANOVA test. *p* < 0.05 indicates the significant difference between EA towards cytokine levels decrease.

### 2.6. Antibacterial Assay

#### 2.6.1. Bacteria

Bacteria used were *Escherichia coli* (ATCC 35281) and *Salmonella enterica* (ATCC 14028). The bacteria were inoculated in nutrient agar (NA) and incubated at 37°C for 24 hrs. Each bacterium was then suspended in 0.9% NaCl to adjust to the turbidity of 0.5 McFarland scale [19].

#### 2.6.2. Broth Microdilution Assay

From stock solution, EA (8192 *μ*g/mL) was diluted into 12 concentrations which were 4096, 2048, 1024, 312, 256, 128, 64, 32, 16, 8, 4, and 2 *μ*g/mL. The diluted concentrations were filled in the wells of microplates' numbers 1–12. It was conducted in duplicate in rows A and B. Rows A-B were filled with EA, bacteria, and media, rows C-D were filled with chloramphenicol, bacteria, and media as the positive control, rows E-F were filled with 10% DMSO, bacteria, and media as the negative control, row G was filled with bacteria and media as normal control, and row H was only filled with media as sterility control. The plates were incubated at 37°C for 24 hrs. The reading of MIC was made by absorbance under a microplate reader [19].

## 3. Results

### 3.1. Phytochemical Screening

The phytochemical screening results can be seen in Table 1. It provides alkaloids, flavonoids, tannins, steroids, and phenols. This result is according to the phytochemical screening of another *Etlingera* genus containing alkaloids, flavonoids, phenolics, and essential oil.

### 3.2. Effect of EA on Carrageenan-Induced Rat Hind Paw Edema

**Figure 1:**
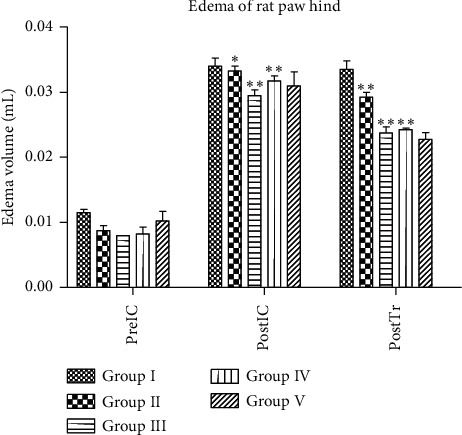
The measurement of palms' edema volume (Group I = negative control, Group II = EA200, Group III = EA300, Group IV = EA400, and Group V = positive control; PreIC = preinduced carrageenan, PostIC = postinduced carrageenan, and PostTr = posttreatment; data are presented as mean ± SEM). ^*∗*^*p* > 0.05 vs. Group I; ^*∗∗*^*p* < 0.05 vs. group I.

As shown in Figure 1, all groups experienced increased edema volume than preIC (preinduced carrageenan) (*p* < 0.05). Compared with posttreatment, the edema volume of Groups III–V was decreased. It showed that Groups III and IV were significant in decreasing the edema volume compared to Group I (*p* < 0.05). They also had similar decreased edema volume to Group V (*p* > 0.05) posttreatment.

### 3.3. Effect of EA on Plasma IL-6, IL-12, and TNF-*α*

**Figure 2:**
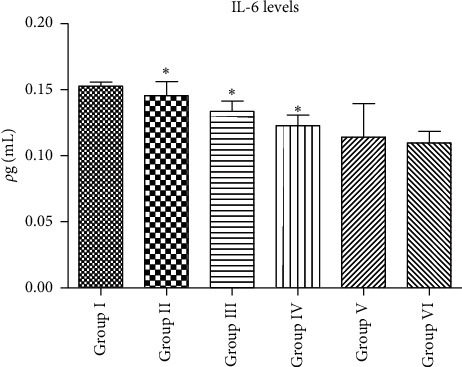
The plasma IL-6 levels (Group I = negative control; Group II = EA200; Group III = EA300; Group IV = EA400; Group V = positive control; and Group VI = normal control; data are presented as mean ± SEM) ^*∗*^*p* > 0.05 vs. Group I; ^*∗∗*^*p* < 0.05 vs. Group I.

Figure 2 demonstrates that Group I had the highest IL-6 levels than Group V (*p* < 0.05). For EA Groups II–IV, they exhibited decreased plasma IL-6 levels in a concentration-dependent manner, yet Group II was not significantly different from Group I (*p* > 0.05). In contrast, Groups III and IV exhibited significantly decreased plasma Il-6 levels (*p* < 0.05). Meanwhile, Groups II–IV were exhibiting a significant difference to Group V, yet providing an anti-inflammatory effect. In conclusion, in Group IV, EA 400 is the optimal concentration in decreasing plasma IL-6 levels.

**Figure 3:**
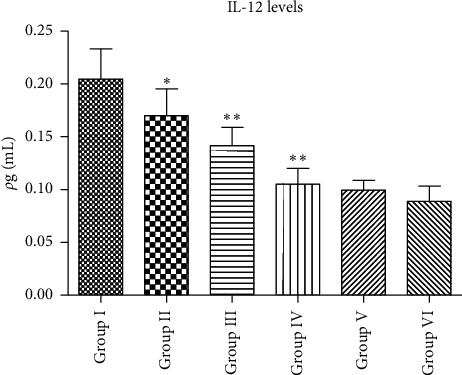
The plasma IL-12 levels (Group I = negative control, Group II = EA200, Group III = EA300, Group IV = EA400, Group V = positive control, and Group VI = normal control; data are presented as mean ± SEM), ^*∗*^*p* > 0.05 vs. Group I; ^*∗∗*^*p* < 0.05 vs. Group I.

Normal group became the group that had the lowest IL-12 levels at 0.08875 *ρ*g/mL (Group VI), while the negative control group showed the highest levels at 0.2045 *ρ*g/mL (Group I) (*p* > 0.05). The EA groups were concentration-dependent; with the highest concentration, the lowest plasma IL-12 levels reduce. The IL-12 levels were 0.17 *ρ*g/mL, 0.1415 *ρ*g/mL, and 0.10525 *ρ*g/mL, respectively. Compared with Group V as the positive control, Groups II and III were significantly different (*p* < 0.05), whereas Group V was not significantly different (*p* > 0.05). EA400 is an optimal concentration as an anti-inflammatory; thus, it has similar activity to the positive control, sodium diclofenac. It is presented in Figure 3.

**Figure 4:**
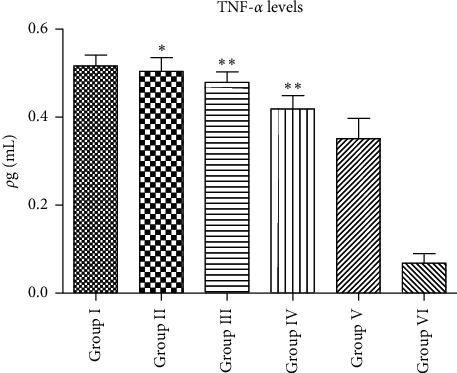
The plasma TNF-*α* levels (Group I = negative control, Group II = EA200, Group III = EA300, Group IV = EA400, Group V = positive control, and Group VI = normal control; data are presented as mean ± SEM), ^*∗*^*p* > 0.05 vs. Group I; ^*∗∗*^*p* < 0.05 vs. Group I.

According to Figure 4, Groups II–IV as EA group showed decreased plasma TNF-*α* levels in a concentration-dependent manner. The plasma TNF-*α* level of EA was 0.504 *ρ*g/mL, 0.479 *ρ*g/mL, and 0.419 *ρ*g/mL, for EA400, EA300, and EA200, respectively. Comparing with Group I, Groups III and IV were significantly different (*p* < 0.05), while Group II was not significantly different in decreasing plasma TNF-*α* levels (*p* > 0.05). In contrast, they were significant to Group V in decreasing plasma TNF-*α* levels. It concluded that EA could decrease the plasma TNF-*α* levels in the inflammatory model, but not effective as Group V as the positive control. The optimal concentration was EA400.

### 3.4. Effect of EA on *E. coli* and *S. enterica*

Table 2 shows that *E. alba* rhizome extract has an antibacterial activity against both bacteria. However, it was weaker than chloramphenicol. The extract still has numerous other compounds, thus affecting its ability to inhibit antibacterial growth.

## 4. Discussion

Phytochemical screening aims to determine the presence of secondary metabolites in the extract [17]. The components in the extract of *E. alba* rhizome analysis were performed using a qualitative method using a test tube test. The test tube is carried out by adding specific reagents to plant extracts to produce a specific solution/precipitate color that indicates a particular compound [20]. The components detected in EA rhizome extract were alkaloids, flavonoids, tannins, steroids, and phenols. These components might be responsible for providing the pharmacological properties of EA rhizome [21, 22].

These data showed that the EA is concentration-dependent, which means that the higher the EA concentration, the higher the effect of reducing edema volume. The optimal concentration of EA to decrease the edema volume and cytokines, namely, IL-6, Il-12, and TNF-*α*, is 400 mg/kg BW. According to a previous study, the EA 400 mg/kg BW dose potentially provides an anti-inflammatory effect [23]. The anti-inflammatory activity of EA extract is probably due to the presence of flavonoids. According to the same study, the flavonoid content caused the anti-inflammatory activity of *E. elatior* extract. However, EA has not provided a better anti-inflammatory effect than the positive control. The diclofenac sodium is a potent anti-inflammatory drug that inhibits inflammatory mediators' formation by inhibiting the COX enzyme.

In particular, flavonoid groups provide an anti-inflammatory effect in numerous pathways, including inhibiting the COX and lipoxygenase enzymes' activity, directly causing the inhibition of prostaglandin leukotriene biosynthesis, which are the end products of the COX and lipoxygenase pathways. In addition, flavonoid inhibits the accumulation of leukocytes and neutrophils' degranulation, thereby directly reducing arachidonic acid release by neutrophils and inhibiting histamine release. In normal conditions, leukocytes move freely along the endothelial wall. During inflammation, various endothelial-derived mediators and complement factors cause adhesion of leukocytes to the endothelial wall as well as triggering the oxidative stress. Administration of flavonoids can reduce the number of leukocytes and the complement activation, thereby decreasing leukocyte adhesion to the endothelium and resulting in a decrease in the body's inflammatory response. The flavonoids and phenolic compounds are also capable of preventing the oxidation [24, 25].

The compounds in EA are thought to inhibit proinflammatory cytokines indirectly so that they can be potentially anti-inflammatory. Flavonoids reduce the secretion of NF-*κ*B and P-1 activities, which play an essential role in modulating proinflammatory mediators such as IL-6, IL-12, and TNF-*α* [26]. Like flavonoids, steroids and alkaloids also inhibit the NF-*κ*B pathway responsible for inflammatory states by inactivating the MAP kinase [27, 28]. It inactivated macrophages that involve upregulation of inflammatory states [29].

The results obtained were compared with positive and negative controls. Positive control is used to determine the effectiveness of the sample tested; if the MIC value of the sample formed is smaller than with the positive control, the sample is more effective as antibacterial as well. The negative control used is DMSO 10% to prove the inertness of the solvent. In contrast, chloramphenicol is positive control, which is a broad-spectrum antibacterial that inhibits both Gram-positive and Gram-negative. It is bacteriostatic at a low dose and bactericidal at high dose [30]. DMSO 10% is used because of this solvent's nature to dissolve polar and nonpolar compounds, where with a concentration of 10%, DMSO cannot inhibit cell growth in bacteria [31]. The MIC test sample of EA extracts with a diluted concentration of 4096 *μ*g/mL to 2 *μ*g/mL.

Antibacterial based on the MIC value is categorized into four: active compounds having a MIC <100 *μ*g/mL as powerful antibacterial activity, between 100 and 500 *μ*g/mL as relatively strong antibacterial activity, between 500 and 1000 *μ*g/mL are as weak antibacterial activity, and >1000 *μ*g/mL as suspected of being antibacterial only [32].

EA is thought to have activity as an antibacterial caused by secondary metabolites that act as antibacterials such as flavonoids, alkaloids, tannins, and terpenoids. That can be the reason of using *E. alba* rhizome as folklore medicine. The mechanism of flavonoids is forming complex compounds with extracellular and dissolved proteins to damage the bacterial cell membrane [33–35]. The alkaloid mechanism inhibiting microbial growth is by disrupting peptidoglycan components in the cell wall; thereby, the cell wall layer is not formed entirely and causes cell death [36]. The terpenoid reacts with porin (protein transmembrane) on the bacterial cell wall's outer membrane, thus forming strong polymer bonds resulting in the breakdown of porin [37]. Tannins inhibit microbial growth by to form irreversible complexes with nucleophilic amino acid; thus, they inactivate the microbial adhesins, enzymes, and transport proteins on cell membranes [38, 39].

## 5. Conclusions

*Etlingera alba* (A.D.) Poulsen provides pharmacological activities, namely, anti-inflammatory at 400 mg/kg BW concentration and antibacterial against *E. coli* and *S. enterica.* The alkaloids, flavonoids, tannins, steroids, and phenols contained in *E. alba* rhizome extract might be playing a vital role in those properties.

## Figures and Tables

**Table 1:** Phytochemical screening of extract *E. alba* rhizome.

Phytochemical screening	Results
Alkaloid	+
Flavonoid	+
Saponin	−
Tannin	+
Terpenoid	−
Steroid	+
Phenol	+

*∗*+ = positive; − = negative.

**Table 2:** MIC value of *E. alba* rhizome extract.

Sample	MIC value (*μ*g·mL)	
	*E. coli*	*S. enterica*
*E. alba* rhizome extract	>64	>32
Chloramphenicol	2	2

## Data Availability

The data used to support the findings of this study are available from the corresponding author upon request.
